# A meteorological-based forecasting model for predicting minimal infection rates in *Culex pipiens-restuans* complex using Québec’s West Nile virus integrated surveillance system

**DOI:** 10.14745/ccdr.v48i05a03

**Published:** 2022-05-05

**Authors:** Julie Ducrocq, Karl Forest-Bérard, Najwa Ouhoummane, Elhadji Laouan Sidi, Antoinette Ludwig, Alejandra Irace-Cima

**Affiliations:** 1 Institut national de santé publique du Québec, Montréal, QC; 2 Scientific Operations and Response, National Microbiology Laboratory, Public Health Agency of Canada, Saint-Hyacinthe, QC

**Keywords:** epidemiology, outbreaks, forecasting tool, Québec, West Nile virus

## Abstract

**Background:**

The *ministère de la Santé et des Services sociaux (MSSS) du Québec* (Québec’s health authority) has expressed an interest in the development of an early warning tool to identify seasonal human outbreaks of West Nile virus infection in order to modulate public health interventions. The objective of this study was to determine if a user-friendly meteorological-based forecasting tool could be used to predict minimal infection rates for the *Culex pipiens-restuans* complex—a proxy of human risk—ahead of mosquito season.

**Methods:**

Annual minimal infection rate (number of positive pools/number of mosquitoes) was calculated for 856 mosquito traps set from 2003 to 2006 and 2013 to 2018 throughout the south of Québec’s. Coefficient of determination (R^2^) were estimated using the validation dataset (one third of the database by random selection) with generalized estimation equations, which were prior fitted backwards with polynomial terms using the training dataset (two thirds of the database), in order to minimize the Bayesian information criteria. Mean temperatures and precipitation were grouped at five temporal scales (by month, by season and by 4, 6 and 10-months groupings).

**Results:**

Mean temperatures and cumulative precipitation from the previous months of March (R^2^=0.37), May (R^2^=0.36), December (R^2^=0.35) and the autumn season (R^2^=0.38) accounted for ~40% of *Cx. pipiens-restuans* annual minimal infection rates variations. Including the “year of sampling” variable in all regression models increased the predictive abilities (R^2^ between 0.42 and 0.57).

**Conclusion:**

All regression models explored have too weak predictive abilities to be useful as a public health tool. Other factors implicated in the epidemiology of the West Nile virus need to be incorporated in a meteorological-based early warning model for it to be useful to the provincial health authorities.

## Introduction

West Nile virus (WNV) has been the most important mosquito-borne infection in Québec for the past two decades (1). A provincial surveillance program specific for this arbovirus has been put in place since 2000 (2), one year after its first detection in North America following an outbreak of neuro-invasive diseases in the state of New York, United States (US) (3). It was composed of an enhanced passive surveillance of humans, of sick or dead wild birds (which act as reservoirs or accidental hosts) and active mosquito surveillance (4).The surveillance system confirmed WNV’s introduction in Québec in 2002 with the first WNV-positive birds and locally-acquired human cases (5,6). Neurological cases of WNV-infections typically represent a very small proportion (~1%) of infected individuals since 70% to 80% of infections remain asymptomatic, while the rest have unspecific symptoms (i.e. influenza-like illness) (3). Between 2003 and 2018, a total of 541 cases (n=24 deaths) were reported in Québec with the following clinical presentation: neurological impairment (70%); non-neurological (23%); asymptomatic (6%); and unknown (1%) (1). Since then, two peaks of reported cases have been observed in 2012 (n=134; 30% of cases from across Canada) and 2018 (n=200; 46% of cases from across Canada) (1,7).Yearly fluctuations in the number of human cases are observed in other regions (8,9) and appeared to be associated with phenomena occurring at different geographic and temporal scales (10) (i.e. according to local interactions between mosquitoes, birds and humans, environmental factors and large-scale climatic fluctuations such as the El Niño-Southern Oscillation) (11–14).Scientists are still working to understand and identify factors influencing outbreaks of WNV infections, and to develop models that would be able to predict when and where these outbreaks could potentially occur. The provincial health authority (*Ministère de la Santé et des Services sociaux*) expressed an interest in the development of an early warning tool to identify outbreak years in advance, and to use this information to mitigate risk and decrease human exposure by modulating their interventions. Such tools are generally based on meteorological data in order to predict a potential outbreak with a delay ranging from a few weeks to a few months before the surveillance system confirms that WNV is circulating (15). Temperature and precipitation are the most commonly used WNV predictors (16). The main goal of this project is to determine if a meteorological-based early warning tool could predict WNV infection rates in mosquitoes.

## Methods

**Figure 1:**
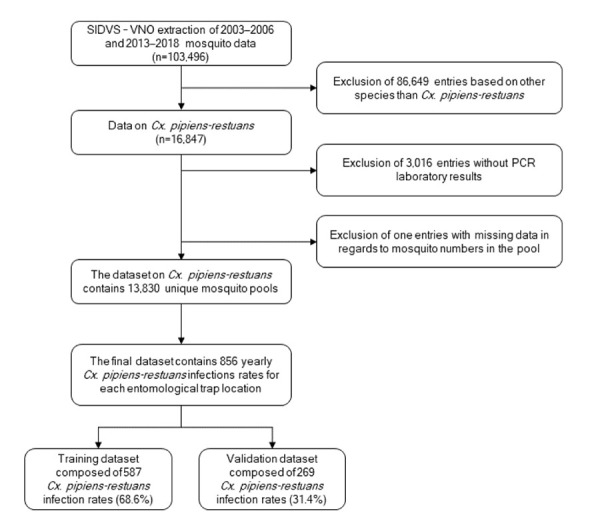
Flow chart of the entomological data extracted from Québec’s *Système intégrée des données de vigie sanitaire du virus du Nil occidental* Abbreviations: *Cx. pipiens-restuans, Culex pipiens-restuans*; PCR, polymerase chain reaction; SIDVS-VNO, *Système intégrée des données de vigie sanitaire du virus du Nil occidental*

**Figure A1:**
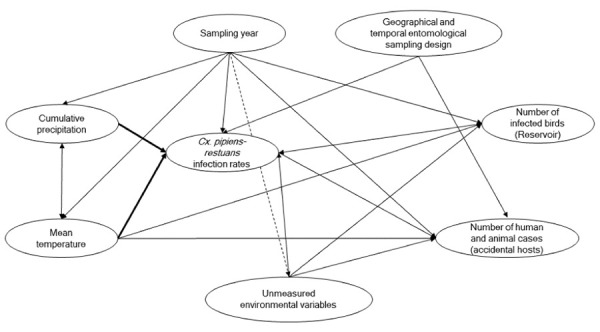
Conceptual framework using a directed acyclic graph between meteorological variables and the main outcomes of Québec’s West Nile virus integrated surveillance system Notes: The darker arrows represent the associations evaluated in this project. To estimate the associations between mean temperatures and precipitations and *Cx. pipiens-restuans* complex minimal infection rates the model needs to be minimally adjusted for the “sampling year” variable. However, since the database already represents the seasonal minimal infection rate for each mosquito trap-year, this variable has not been directly included in the regression models of the principal analysis. Its inclusion was rather evaluated using a sensitivity analysis

The history of Québec’s WNV integrated (using human, animal and entomological components) surveillance system is fully described elsewhere (17). Background screening of the scientific literature was used to build a directed acyclic graph (DAG) (**Figure A1**) to conceptualize relationships between potential drivers of WNV and identify important variables to include in models, using the DAGitty tool (18).Data from 13,830 mosquito pools of the *Culex pipiens-restuans* complex, these two species espèces (*Cx. pipiens* and *Cx. restuans*) cannot be differentiate morphologically (19), were extracted from the *Système intégrée des données de vigie sanitaire du virus du Nil occidental* (20) to calculate annual minimal infection rate (MIR) (number of positive pools/number of mosquitoes in each pool) (**Figure 1**). The MIRs were calculated for each of the 856 mosquito pools, which were grouped for each trap deployed per year (i.e. mosquito trap-year) and distanced by greater or equal to 1 km^2^ radius, since it represents the average travel distance of *Cx. pipiens-restuans* (21) and the spatial resolution of the meteorological data (1 km^2^). Mosquito surveillance was carried out inconsistently in 12 out of 18 Public Health Units (PHU), with a greater number of mosquito pools tested for Montreal and Montérégie (**Table 1**), except since 2017 where 49 traps are deployed annually in the same seven PHU (Montréal=5, Laval=4, Montérégie=5, Outaouais=7, Lanaudière=7, Mauricie-Centre-du-Québec=7 et Capitale-Nationale=14).

**Table 1:** Descriptions of *Culex pipiens-restuans* minimal infection rates and meteorological data at different geographical and temporal scales

Spatio-temporal variables	Number of data entry	Minimal infection rate per 1,000 mosquitoes		Mean temperatures at mosquito traps		Mean precipitation at mosquito traps	
		n	95% CI	n	min; max	n	min; max
Province of Québec	856	3.1^a^	2.2; 4.0^a^	6.3	2.1; 8.2	3.0	2.2; 4.3
Public Health Units							
01 – Bas Saint-Laurent	0	–	–	–	–	–	–
02 – Saguenay – Lac Saint-Jean	6	0	0; 45.9	2.5	2.1; 2.9	2.6	2.4; 2.8
03 – Capitale Nationale	40	4.4	1.5; 7.3	5.0	3.5; 7.8	3.5	2.9; 4.3
04 – Mauricie et Centre-du-Québec	28	1.6	0; 4.0	5.4	4.4; 7.2	3.1	2.3; 3.5
05 – Estrie	8	0	0; 36.9	5.8	5.0; 7.0	3.5	3.0; 3.8
06 – Montréal	265	3.1	2.2; 3.9	6.4	5.7; 8.2	3.0	2.4; 3.9
07 – Outaouais	47	3.1	0; 7.3	6.0	3.5; 7.8	2.9	2.2; 3.9
08 – Abitibi-Témiscamingue	3	0	0; 70.8	3.2	3.1; 3.3	2.7	2.7; 2.8
09 – Côte-Nord	0	–	–	–	–	–	–
10 – Nord-du-Québec	0	–	–	–	–	–	–
11 – Gaspésie-îles-de-la-Madeleine	0	–	–	–	–	–	–
12 – Chaudières-Appalaches	8	0	0; 36.9	4.6	3.7; 6.2	3.1	2.7; 3.6
13 – Laval	82	4.9	3.1; 6.7	6.5	5.7; 8.0	3.0	2.5; 4.0
14 – Lanaudière	36	3.5	0.6; 6.5	6.7	5.4; 7.8	3.1	2.3; 4.0
15 – Laurentides	70	4.8	2.0; 7.7	6.4	5.3; 8.0	3.2	2.6; 4.2
16 – Montérégie	263	3.9	2.9; 5.0	6.5	5.3; 8.1	2.9	2.4; 3.9
17 – Nunavik	0	–	–	–	–	–	–
18 – Terres-Cries-de-la-Baie-James	0	–	–	–	–	–	–
Type 3 global analysis: Chi-square (*p*-value)^b^	–	9.9	0.5	23.3	0.02	17.2	0.1
Year							
2003	118	3.0	1.7; 4.4	5.5	3.1; 6.3	2.6	2.3; 3.4
2004	86	0.9	0; 2.1	5.7	2.1; 6.5	3.1	2.4; 3.8
2005	146	3.4	2.0; 4.8	6.5	3.7; 7.0	3.1	2.4; 4.3
2006	65	0.2	0; 0.4	7.5	6.8; 7.9	3.8	3.1; 4.2
2007	0	–	–	–	–	–	–
2008	0	–	–	–	–	–	–
2009	0	–	–	–	–	–	–
2010	0	–	–	–	–	–	–
2011	0	–	–	–	–	–	–
2012	0	–	–	–	–	–	–
2013	63	5.5	3.2; 7.8	7.1	6.4; 7.5	2.8	2.4; 3.1
2014	199	4.2	2.7; 5.6	6.0	5.7; 6.2	3.0	2.8; 3.2
2015	45	5.6	2.6; 8.7	6.2	4.5; 6.5	2.7	2.2; 3.4
2016	47^c^	5.6	2.3; 9.0	7.0	3.1; 8.2	3.0	2.5; 3.8
2017	46^c^	5.4	3.2; 7.7	6.5	5.1; 7.7	3.5	3.2; 3.9
2018	41^c^	11.8	0; 91.1	6.2	4.7; 7.5	3.1	2.6; 3.5
Type 3 global analysis: Chi-square (*p*-value)^b^	–	28.5	0.008	61.1	<0.0001	53.9	<0.0001
Month^d^							
January	0	–	–	-10.8	−19.7; −4.2	2.0	0.6; 5.3
February	0	–	–	-8.8	−16.9; −4.1	2.1	0.9; 5.4
March	0	–	–	-3.8	−7.3; 0.5	2.0	0.8; 5.0
April	0	–	–	5.4	−3.0; 7.8	3.6	0.2; 7.4
May	96	0	0; 37.7	13.4	7.5; 16.6	3.2	0.7; 6.8
June	1,518	0	0; 0.1	18.7	13.1; 21.4	4.2	1.5; 7.6
July	3,611	0.8	0.4; 1.2	21.1	18.0; 23.5	3.2	1.6; 7.2
August	4,688	5.5	4.3; 6.7	20.1	16.4; 22.3	3.5	1.7; 6.2
September	3,675	6.0	4.4; 7.5	16.1	12.4; 18.6	2.9	0.8; 7.7
October	242	1.9	0; 4.4	9.0	3,4; 12.9	3.4	1.2; 6.8
November	0	–	–	1.4	−2.3; 4.4	3.0	0.3; 5.6
December	0	–	–	−6.5	−11.8; 1.8	3.2	0.6; 7.1
Type 3 global analysis: Chi-square (*p*-value)^b^	–	15.6	<0.0001	N/A^e^	N/A^e^	N/A^e^	N/A^e^

Abbreviations: CI, confidence interval; N/A, not applicable; –, no information is available

^a^ Minimal infection rates using mosquitoes trapped during the month of August and September was 5.0 (95% CI; 2.6–7.4)

^b^ Using a linear regression model with a repeated measure on the mosquito trap name

^c^ Monthly minimal infection rates were calculated using the dataset containing the 13,830 mosquito pools while the mean monthly temperatures and precipitation were calculated using the 856 mosquito pools grouped by unique trap-year

^d^ These numbers are lower than the fixed number of 49 mosquito traps that have been deployed since 2017, because entries from traps within a 1 km^2^ radius were merged together

^e^ Not available because of multicollinearity (VIF>2.5) in the models

**Figure 2:**
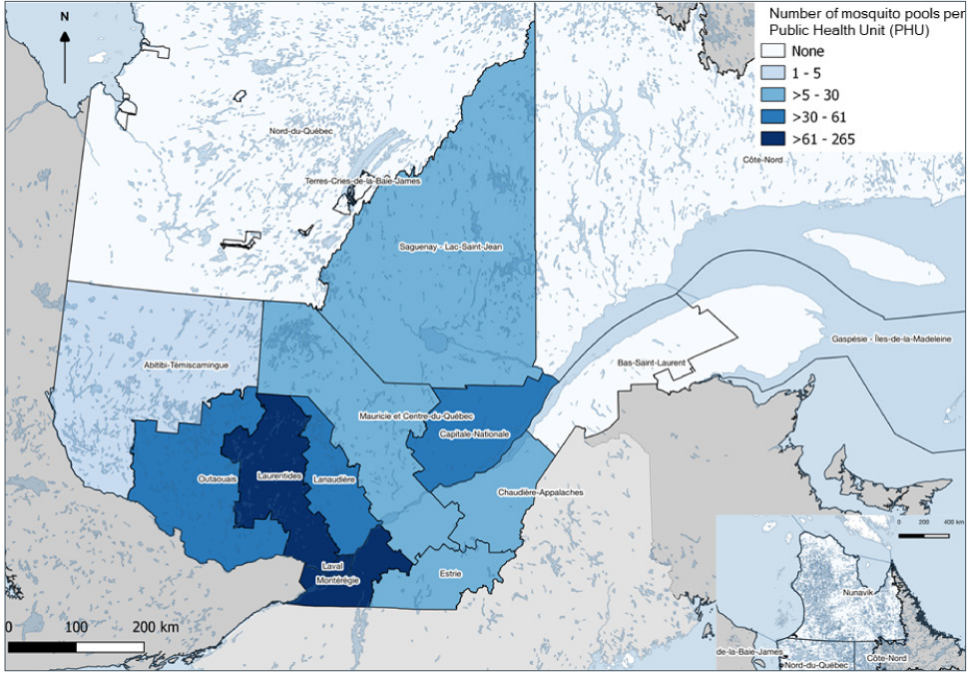
Map of the 18 Public Health Units in the province of Québec according to the number of mosquito pools tested between 2003–2006 and 2013–2018 Note: Created on the 19th of May 2021 with QGIS. Projection NAD83 / MTQ Lambert. Data downloaded from https://donneesquebec.ca/ and https://publications.msss.gouv.qc.ca/msss/document-001647/

Explanatory variables included the mean daily temperatures (T_mean_=T_maximal_+T_minimal_/2) (°C) and daily precipitation (mm) extracted from the Oak Ridge National Laboratory Distributed Active Archive Center (https://daymet.ornl.gov) according to the Global Positioning System (GPS) coordinates of each trap-year (latitude and longitude). Mean daily temperature and sum of precipitation were grouped according to five different temporal scales: 1) previous months; 2) previous seasons: summer (June and July), spring (March to May), winter (December to February) and autumn (September to November); 3) previous four-months (November of the year before to February of the same year); 4) previous six-months (September of the year before to February of the same year); and 5) previous ten-months (September of the previous year to July of the current year) groupings, based on the literature suggesting that meteorological conditions impact WNV transmission months ahead (11,22). Meteorological data from the closest neighboring traps were assigned to eight mosquito traps with missing data, with half of these traps being separated by more than one kilometer from their matched trap. To consider variations in the geographical and temporal mosquito sampling, two new “sampling strengths” variables were created for each PHU (**Figure 2**) based on the number of tested mosquito pool: 1) annually; and 2) during the 2003–2018 period (Table 1).

Bivariate analysis between *Cx. pipiens-restuans* annual MIR (outcome) and explanatory variables (PHU, year and month) were performed using a linear regression model; an auto-regressive working covariance matrix with mosquito traps in the same city were nested within each PHU. Then multivariate regression models evaluated the predictive ability of each temperature and precipitation temporal scale to estimate MIRs, with observations randomly separated into training (n=2/3) and validation (n=1/3) datasets. A backward variable selection with a maximum of four polynomial terms to obtain the lowest Bayesian information criteria using the training dataset was fitted back into a Poisson regression model using the validation dataset (23). MIRs were obtained using the number of positive *Cx. pipiens-restuans* pools on the logarithm of the number of mosquitoes tested, while mosquito traps in the same city were nested within each PHU to account for correlated data using an auto-regressive working covariance matrix. The methodology of sensitivity and post hoc analyses are described in the Annex material. The SAS macro (%RsquareV) was used to calculate the coefficient of determination (R^2^), reflecting the proportion of the variance explained by the predictors (24). Only R^2^≥0.30 are highlighted in the text. The 95% confidence intervals (CI) and *p*-values are presented, and all postulates associated with the regression models were visually inspected (25). Statistical analysis were performed using version 9.4 of SAS/STAT^TM^ software (SAS Institute, Cary, North Carolina, US).

## Results

Since 2003, 172,094 *Cx. pipiens-restuans* mosquitoes were analyzed by polymerase chain reaction (PCR), resulting in a mean MIR of 3.1 per 1,000 mosquitoes (95% CI; 2.2–4.0), when data were grouped under the 856 mosquito trap-years, which varied according to the sampling year (χ^2^=28.5; *p=*0.0008) and month (χ^2^=15.6; *p*<0.0001) but not across the PHU (χ^2^=9.9; *p=*0.5) (Table 1). For the studied period, mean monthly temperatures near mosquito traps varied from 23.5°C (July) to −19.7°C (January) while monthly cumulative precipitation varied from 0.3 mm (November) to 7.7 mm (September).The predictive ability of the previous mean temperatures and precipitation to predict the seasonal MIRs was the highest using the previous months of March (R^2^=0.37), May (R^2^=0.36) and December (R^2^=0.35) as well as the previous autumn season (R^2^=0.38), with all other coefficients of determination below 0.30 (**Table 2**). The results of all the sensitivity and post hoc analysis are fully described in the Annex material. Briefly, including the year of sampling variable in the main regression models increased all R^2^ (range: 0.42 to 0.57) while the increased was less substantial when including the PHU variable (range: 0.18 to 0.46) (**Table A1**).

**Table 2:** The ability of meteorological variables to predict *Culex pipiens-restuans* minimal infection rates, at different temporal grouping scales for the meteorological data

Regression models and equations from the training dataset		Coefficient of determination of the validation dataset (*Penalised R^2^*)
A) By previous month		
July	MIR = -915.67 + 130.81Temp - 6.45Temp^2^ + 0.11Temp^3^ + 35.05Prec – 14.65Prec^2^ + 2.58Prec^3^ – 0.17Prec^4^ + ε	0.28
June^a^	MIR = -40.79 + 3.54Temp – 0.10Temp^2^ + 0.95Prec – 0.09Prec^2^ + ε	0.15
May^a^	MIR = 13.60 – 2.13Temp + 0.09Temp^2^ – 7.98Prec + 2.48Prec^2^ – 0.24Prec^3^ + ε	0.36
April	MIR = -7.64 – 0.27Temp + 2.47Prec – 0.69Prec^2^ + 0.08Prec^3^ – 0.003Prec^4^ + ε	0.19
March	MIR = -22.03 – 4.28Temp – 2.31Temp^2^ - 0.44Temp^3^ – 0.03Temp^4^ + 30.83Prec – 22.71Prec^2^ + 6.79Prec^3^ – 0.70Prec^4^ + ε	0.37
February	MIR = -2.85 + 2.38Temp + 0.20Temp^2^ – 0.01Temp^3^ + ε	0.10
January	MIR = -47.75 – 17.42Temp – 2.65Temp^2^ - 0.18Temp^3^ – 0.004Temp^4^ + 0.34Prec – 0.04Prec^2^ + ε	0.24
December	MIR = -6.93 + 0.21Temp – 0.05Temp^2^ – 0.01Temp^3^ + 2.67Prec – 0.79Prec^2^ – 0.06Prec^3^ + ε	0.35
November	MIR = -4.74 – 0.07Temp – 0.46Temp^2^ + 0.12Temp^3^ + 2.61Prec + 2.74Prec^2^ – 0.65Prec^3^ + 0.09Prec^4^ + ε	0.18
October	MIR = -7.48 + 0.20Temp – 0.83Prec + 0.47Prec^2^ + 0.06Prec^3^ + ε	0.28
September	MIR = -6.02 + 0.16Temp – 1.75Prec + 0.26Prec^2^ + ε	0.28
August	MIR = -13.09 + 0.40Temp – 0.24Prec + ε	0.08
B) By season^b^		
Summer^a^	MIR = -45.53 + 4.37Temp – 0.11Temp^2^ – 3.25Prec + 0.46Prec^2^ + ε	0.15
Spring^c^	MIR = -206.81 + 84.33Temp – 25.56Temp^2^ + 3.36Temp^3^ – 0.16Temp^4^ + 132.67Prec – 64.01Prec^2^ + 13.26Prec^3^ – 1.00Prec^4^ + ε	0.09
Winter	MIR = 2.31 + 0.89Temp + 0.05Temp^2^ – 6.40Prec + 3.04Prec^2^ – 0.46Prec^3^ + ε	0.24
Autumn	MIR = -438.86 + 153.19Temp – 17.32Temp^2^ + 0.65Temp^3^ – 15.35Prec + 4.79Prec^2^ – 0.50Prec^3^ + ε	0.38
C) From November of the previous year to February of the same year		
Four months grouped together	MIR = -48.07 – 2.87Temp – 1.24Temp^2^ – 0.18Temp^3^ – 0.01Temp^4^ + 54.97Prec – 26.15Prec^2^ + 15.35Prec^3^ – 0.41Prec^4^ + ε	0.24
D) From September of the previous year to February of the same year		
Six months grouped together	MIR = 9.37 + 0.48Temp – 0.23Prec + 13.46Prec^2^ – 3.51Prec^3^ + 0.33Prec^4^ + ε	0.27
E) From September of the previous year to July of the same year^c^		
Ten months grouped together	MIR = -119.02 – 113.69Temp – 41.82Temp^2^ + 6.68Temp^3^ – 0.39Temp^4^ + ε	0.12

Abbreviations: MIR, minimal infection rates; Prec, precipitations; Temp, temperature

^a^ A polynomial equation with three terms was used as the model did not converge with four

^b^ Summer represents the months of June and July; Spring represents the months of March to May; Winter represents the months of December to February; Autumn represents the months of September to October

^c^ The repeated measure was limited to each mosquito pool to allow the model to converge as it was not possible to nest mosquito traps from the same city within each Public Health Unit to account for their proximity

## Discussion

This is the first time that a meteorological-based early warning tool to predict *Cx. pipiens-restuans* annual MIRs in Québec has been investigated using the province’s own WNV surveillance data. The ability of mean temperatures and precipitation to predict MIRs was highest when the data were grouped under the previous months of March, December and May, and previous autumn season (September to November of the previous year). However, the predictive capacity of the model is probably too weak to be useful for an early warning system. The fact that only ~40% of the variance in *Cx. pipiens-restuans* annual MIRs can be explained by mean temperature and precipitation suggests that others factors are implicated in the epidemiology of WNV and that these factors need to be incorporated into this kind of predictive tool (15).Many early warning or forecasting systems that are primarily or solely based on meteorological data have been developed to predict other mosquito-borne diseases (i.e. chikungunya, dengue, malaria, yellow fever, Zika) to anticipate vector control responses (24). Our results suggest that presently these models yield insufficient predicting abilities to be useful in real life for Quebec’s public health authority. The epidemiology of WNV is known to be complex and adding variables such as environmental drivers (habitat suitability or distribution of vectors and reservoirs, vegetation index, land use) and data on avian hosts (abundance, migrations) usually improve predictions (15). However, the necessity of integrating data from the ongoing surveillance system and refining the geographical scale leads to less user-friendly tools, for public health authorities (26).

### Strengths and limitations

One of the strengths of this project is that the geographical proximity of mosquito traps was accounted for in most of the regression models, as this can bias results. Additionally, multiple sensitivity and post hoc analyses brought robustness to our results. The polynomial equations accounted for non-linear associations between meteorological variables and MIRs, allowing for a better representation of their relationship and a better model fit, while the backward selection of variables proved to be as effective as the visual inspection of generalized additive models. Because MIRs are low numerical values, an attempt to increase the statistical power was made by calculating annual MIRs using mosquitoes trapped during August and September. Though the mean annual MIRs increased for most positive mosquito pools, the coefficient of determination for most regression models decreased unexpectedly; possibly as a consequence of a smaller sample size when including only August and September, since 13 mosquito traps had no mosquitoes captured, which decreased statistical power.Based on our conceptual framework, it is unknown if an over adjustment bias occurred when adjusting for “year of sampling” in the regression models (27). Despite multiple sensitivity analysis with potential confounding variables, a residual confounding effect cannot be discarded (28). Because mosquito pools were grouped for each trap-year, it was not possible to account for the different types of traps that were employed. This limitation has probably a marginal effect, since 85.2% of mosquitoes were captured by the CDC light traps. It was not possible to account for the use of larvicides during the 2003–2005 and 2013–2014 seasons, which might have influenced the estimates. Because mosquito PCR testing is done independently from mosquito trapping, if a measurement bias was to be present, a decrease in the association would also be suspected.A selection bias could influence our results because of the geographical distribution of traps which is linked to 1) past human WNV incidence and 2) the influence of weather on human physical activity and exposure to mosquitoes (29,30). Additionally, the fixed location of mosquitoes traps since 2017 comes at the expense of detecting WNV emergence in the less densely populated PHU situated at more northern latitudes. The limited number of mosquito traps on such a huge territory and the absence of mosquito surveillance during the colder months reduces the statistical power. Hence, a higher number of traps distributed over a wider geographical area would provide a wider range of meteorological data and increase statistical power. Since WNV vectors vary according to regions, and transmission pathways seem to be influenced environmental and geographical variables at a finer scale then the PHU results of this project may not be transferable to other situations. Despite these limitations, Québec’s WNV surveillance data will be explored further. The next steps will be to directly predict human WNV incidence using meteorological data and bypass the gap that occurred in entomological data between 2007 and 2012 and to explore the development of an early detection system tool.

## Conclusion

All regression models explored have too weak predictive abilities to be useful as a public health tool. Other factors implicated in the epidemiology of the West Nile virus need to be incorporated in a meteorological-based early warning model for it to be useful to the provincial health authorities.

## Annex

### Methods

Because human cases of West Nile virus (WNV) infection are usually reported at the beginning of August (1) and that the objective is to develop an early alert tool able to predict these human cases a couple of months before they occur, temperatures and precipitation during August have not been included in our analysis.

Background screening of the scientific literature was used to build a directed acyclic graph (DAG) (**Figure A1**) to conceptualize relationships between potential drivers of WNV and identify the variables that need to be adjusted for in the model using the DAGitty tool.

#### Sensitivity and post hoc analysis

Seven sensitivity analyses were carried out as follows: 1) regression models were fitted using visual inspection of graphs generated by a generalized additive model with a maximum of four degree of smoothing performed by generalized cross validation; 2) the main models were reanalyzed without the data from the four mosquito traps (LAV 034, LAV 904, SHA 002 and WEN 001) that inherited meteorological data of their closest neighbour with a radius superior to 1 km^2^; 3) by including the Public Health Units (PHU) 2003–2018 sampling strength variable; 4) by including the annual sampling strength by PHU; 5) by attributing the minimal infection rate of mosquitoes trapped only during the month of August and September instead of during the whole summer; 6) by adding the year of sampling in the model; and 7) by adding the PHU in the model instead of in the repeated measure. Following results from the 6^th^ sensitivity analysis, year of sampling was used as a proxy for unmeasured confounders. An post hoc Poisson analyses was performed by modeling directly the MIR using the year of sampling (categorical) as the explanatory variable. To measure if the meteorological variables were confounders of this association, the year of sampling was included as a continuous variable to facilitate the calculation and interpretation of the difference and ratio between both estimates (β_Year of sampling_). Confounding was present based on a difference and/or ratio of estimates superior to 0.1 decimal or 10% (31). Four additional bivariate analysis were carried out with the year of sampling as an explanatory continuous variable and respectively, the mean annual temperatures, mean annual precipitation, annual sampling strength and PHU sampling strength as the main outcome. Specifying a normal distribution, an “identity” link and an auto-regressive working covariance matrix with mosquito traps in the same city nested within each PHU, yielded estimates representing differences in minimal infection rates (MIRs).

### Results

#### Sensitivity analysis

Fitting regression models after a visual inspection of the generalized additive model graphs led to similar results of the main analysis, except for the month of February (R^2^ increased to 0.22) and August (R^2^ increased to 0.24) as well as the ten-month grouped (R^2^ increased to 0.18), while all other comparisons had difference within five decimal units (**Table A1**). Excluding the four entomological traps with meteorological data borrowed from the closest neighboring trap influenced the results for the months of May (R^2^ decreased to 0.19) and September (R^2^ increased to 0.38), summer (R^2^ increased to 0.29) and winter (R^2^ decreased to 0.16) (Table A1). When including the PHU sampling strength variable, all R^2^ were within five decimals of the main results (Table A1). Including the variable reflecting the annual strength of sampling, increased the predictive abilities of at least five decimals for nine temporal groupings (June, April, February, November, August; summer and spring; the six and ten-months groupings). Using the minimal infection rates of *Cx. pipiens-restuans* trapped in August and September instead of the whole summer, influenced the R^2^ for 14 temporal groupings (decreased for May, April, December, November, September, summer, winter, autumn, four and six-months grouping and increased for June, October, August and spring). When including the year of sampling in the main regression models, all coefficient of determination increased between 0.42 and 0.57. Including the PHU variable in the main regression models increased the predictive ability of at least five decimals for all temporal groupings except July, May and autumn.

**Table A1:** Penalized coefficients of determination (Penalized R^2^ for all seven sensitivity analyses)

Regression models	Sensitivity analyses^a^						
	Using generalized additive models for model specifications	Without the four entomological traps	Including the PHU strength of sampling variable	Including the annual sampling strength variable	Using the August and September MIR	Adding the year of sampling in the main model	Adding the PHU variable in the main model
A) By previous month							
July	0.28	0.28	0.27	0.30	0.31	0.56 ↑	0.29
June^b^	0.18	0.15	0.17	0.21 ↑	0.24 ↑	0.53 ↑	0.24 ↑
May^b^	0.36	0.19 ↓	0.35	0.39	0.30 ↓	0.53 ↑	0.35
April	0.23	0.19	0.19	0.31 ↑	0.13 ↓	0.55 ↑	0.31 ↑
March	0.37	0.37	0.38	0.34	0.41	0.53 ↑	0.46 ↑
February	0.22 ↑	0.22 ↑	0.10	0.20 ↑	0.09	0.53 ↑	0.22 ↑
January	0.26	0.24	0.24	0.27	0.21	0.57 ↑	0.30 ↑
December	0.33	0.34	0.37	0.36	0.29 ↓	0.53 ↑	0.41 ↑
November	0.18	0.18	0.19	0.33 ↑	0.04 ↓	0.54 ↑	0.36 ↑
October	0.28	0.28	0.27	0.30	0.34 ↑	0.51 ↑	0.41 ↑
September^b^	0.29	0.38 ↑	0.30	0.35 ↑	0.21 ↓	0.57 ↑	0.38 ↑
August	0.24 ↑	0.11	0.09	0.23 ↑	0.24 ↑	0.48 ↑	0.20 ↑
B) By season^c^							
Summer	0.14	0.29 ↑	0.15	0.24^d^ ↑	0.02 ↓	0.55 ↑	0.27^e^ ↑
Spring	0.09	0.09	0.09	0.18 ↑	0.15^e^ ↑	0.53 ↑	0.23^e^ ↑
Winter	0.20	0.16 ↓	0.23	0.26	0.08↓	0.51 ↑	0.32 ↑
Autumn	0.38	0.38	0.38	0.40	0.27 ↓	0.53 ↑	0.41
C) Four months grouping							
From November of the year before to February of the same year	0.26	0.24	0.24	0.23	0.14 ↓	0.57 ↑	0.35 ↑
D) Six months grouping							
From September of the year before to February of the same year	0.27	0.26	0.29	0.34 ↑	0.19 ↓	0.56 ↑	0.36 ↑
E) Ten months grouped together^b,d^							
From September of the year before to July of the same year	0.18 ↑	0.12^e^	0.13^e^	0.20 ↑	0.17	0.42 ↑	0.18 ↑

Abbreviations: MIR, minimal infection rates; PHU, public health unit

^a^ Arrows indicate the direction of change above and below five decimal units compared to results of the main analysis in Table 2

^b^ A polynomial equation with three terms was used as the model did not converge with four

^c^ Summer represents the months of June and July; Spring represents the months of March to May; Winter represents the months of December to February; Autumn represents the months of September to October

^d^ The repeated measure was limited to each mosquito pool to allow the model to converge as it was not possible to nest mosquito traps from the same city within each Public Health Unit

^e^ No repeated measure was employed for this model to converge

#### Post hoc analysis

When modelling directly the MIR using only the “year of sampling” variable, the R^2^ was 0.53. The estimate associated with the year of sampling on a continuous linear scale and as the only explanatory variable of *Cx. pipiens-restuans* MIRs was 1.09 (95% CI; 1.06–1.11; *p*<0.0001) while yearly fluctuations are observed when treated on the categorical scale (results not shown). The results of the estimates obtained in all the main regression models using the meteorological variables and year of sampling varied between 1.06 and 1.15, with very low confounding effects on the difference and ratio scales (**Table A2**). When comparing MIRs between years and using 2018 as the reference, all anterior sampling years had lower values except for 2016 since the upper 95% CI of the estimate overlaps the null value (**Table A3**). When the year of sampling is treated on a continuous scale, the number of mosquito traps decreased linearly from 2003 to 2018 (β=−1.13 [−1.45–−0.81; *p*<0.0001]). Therefore, the number of mosquito traps and MIRs are inversely correlated between 2003 and 2018 with an increase of 2% in MIRs for each less mosquito trap in time (β=0.98 [0.98–0.99; *p*<0.0001]). At the level of each mosquito trap location in Québec, mean annual temperatures increased (β=0.04 [0.02–0.06; *p*=0.0003]) while mean annual precipitation decreased (β=−0.008 [−0.02–0.006; *p*=0.07]) from 2003 to 2018.

**Table A2:** Differences and ratios between estimates for sampling year in the model, with and without meteorological data using the 2003–2018 entomological dataset

Regression models	Estimate for the sampling year as a continuous variable with meteorological data		*p*-value	Difference in estimates^a^	Ratio in estimates for the sampling year^b^
	n	95% CI			
A) By previous month					
July	1.08	1.04–1.12	<0.0001	0.01	1.01
June^c^	1.11	1.07–1.15	<0.0001	−0.02	0.98
May^c^	1.09	1.06–1.12	<0.0001	0.00	1.00
April	1.07	1.04–1.11	0.0001	0.02	1.02
March	1.15	1.12–1.18	<0.0001	−0.06	0.95
February	1.08	1.05–1.11	<0.0001	0.01	1.01
January	1.14	1.10–1.18	<0.0001	−0.05	0.96
December	1.11	1.06–1.14	0.0007	−0.02	0.98
November	1.06	1.03–1.09	0.0003	0.03	1.03
October	1.08	1.04–1.13	<0.0001	0.01	1.01
September	1.09	1.06–1.13	<0.0001	0.00	1.00
August	1.09	1.07–1.12	<0.0001	0.00	1.00
B) By season^d^					
Summer^c^	1.12	1.10–1.15	<0.0001	−0.03	0.97
Spring^e^	1.09	1.06–1.1	<0.0001	0.00	1.00
Winter	1.12	1.07–1.16	<0.0001	−0.03	0.97
Autumn	1.08	1.05–1.11	<0.0001	0.01	1.01
C) Four months grouping					
From November of the year before to February of the same year	1.09	1.05–1.13	<0.0001	0.00	1.00
D) Six months grouping					
From September of the year before to February of the same year	1.09	1.06–1.11	<0.0001	0.00	1.00
E) Ten months grouping^f^					
From September of the year before to July of the same year	1.08	1.06–1.10	<0.0001	0.01	1.01

^a^ β without meteorological data equivalent to 1.09 (95% CI; 1.06–1.12; *p*-value <0.0001) – β with meteorological data

^b^ β without meteorological data equivalent to 1.09 (95% CI; 1.06–1.12; *p*-value <0.0001) / β with meteorological data

^c^ A polynomial equation with three terms was used as the model did not converge with four

^d^ Summer represents the months of June and July; Spring represents the months of March to May; Winter represents the months of December to February; Autumn represents the months of September to October

^e^ The repeated measure was limited to each mosquito pool to allow the model to converge as it was not possible to nest mosquito traps from the same city within each Public Health Unit

^f^ No repeated measure was employed for models to converge

**Table A3:** Results of the post hoc regression model using the year of sampling to predict *Cx. pipiens-restuans* minimal infection rates using the 2003–2018 entomological dataset

Year of sampling	*Cx. pipiens-restuans* minimal infection rates per 1,000 mosquitoes		Minimal infection rate ratio		*p*–value
	n	Rate	n	Rate	
2003	3.0	1.7–4.4	0.24	0.16–0.34	<0.0001^a^
2004	0.9	0–2.1	0.06	0.04–0.10	<0.0001^a^
2005	3.4	2.0–4.8	0.21	0.12–0.36	<0.0001^a^
2006	0.2	0–0.4	0.05	0.03–0.10	<0.0001^a^
2013	5.5	3.2–7.8	0.41	0.26–0.66	0.0002^a^
2014	4.2	2.7–5.6	0.31	0.20–0.46	<0.0001^a^
2015	5.6	2.6–8.7	0.36	0.21–0.65	0.0005^a^
2016	5.6	2.3–9.0	0.50	0.22–1.13	0.1
2017	5.4	3.2–7.7	0.53	0.35–0.80	0.002^a^
2018 (Reference)	11.8	0–91.1	1.00	1.00	–
Type 3 global analysis (with 9 degree of liberty)	–	–	Χ^2^=17.98	Χ^2^=17.98	0.04

Abbreviation: –, information not available

^a^ Statistically significant after the Bonferroni correction for nine comparisons using a threshold of 0.006 (corresponding to 0.05/9)
